# Caffeine-Related Deaths: Manner of Deaths and Categories at Risk

**DOI:** 10.3390/nu10050611

**Published:** 2018-05-14

**Authors:** Simone Cappelletti, Daria Piacentino, Vittorio Fineschi, Paola Frati, Luigi Cipolloni, Mariarosaria Aromatario

**Affiliations:** 1 Department of Anatomical, Histological, Forensic Medicine and Orthopedic Sciences, Sapienza University of Rome, 00161 Rome, Italy; simone.cappalletti@uniroma1.it (S.C.); paola.frati@uniroma1.it (P.F.); luigi.cipolloni@uniroma1.it (L.C.); mariarosaria.aromatario@uniroma1.it (M.A.); 2 NESMOS (Neuroscience, Mental Health, and Sensory Organs) Department, Sapienza University of Rome, 00161 Rome, Italy; daria.piacentino@uniroma1.it

**Keywords:** accidental death, caffeine, caffeine intoxication, intoxication, Suicide

## Abstract

Caffeine is the most widely consumed psychoactive compound worldwide. It is mostly found in coffee, tea, energizing drinks and in some drugs. However, it has become really easy to obtain pure caffeine (powder or tablets) on the Internet markets. Mechanisms of action are dose-dependent. Serious toxicities such as seizure and cardiac arrhythmias, seen with caffeine plasma concentrations of 15 mg/L or higher, have caused poisoning or, rarely, death; otherwise concentrations of 3–6 mg/kg are considered safe. Caffeine concentrations of 80–100 mg/L are considered lethal. The aim of this systematic review, performed following the Preferred Reporting Items for Systematic Review and Meta-Analyses (PRISMA) statement for the identification and selection of studies, is to review fatal cases in which caffeine has been recognized as the only cause of death in order to identify potential categories at risk. A total of 92 cases have been identified. These events happened more frequently in infants, psychiatric patients, and athletes. Although caffeine intoxication is relatively uncommon, raising awareness about its lethal consequences could be useful for both clinicians and pathologists to identify possible unrecognized cases and prevent related severe health conditions and deaths.

## 1. Introduction

In recent years, the risk of caffeine intoxication has increased due to the more widespread availability of analgesics, CNS stimulant medicine and dietary supplements at shops, health stores and e-markets. Nonetheless, lethal cases from caffeine intoxication are quite uncommon. The first paper about lethal caffeine intoxication was published by Jokela et al. in 1959 [1], and it described the accidental death of a young woman following intravenous administration of caffeine.

The pharmacological effects of caffeine include central nervous system and cardiac stimulation and usually occur at plasma concentrations of 15 mg/L or higher. Common features of caffeine intoxication, also known as “caffeinism” (i.e., a state of chronic toxicity from excessive caffeine consumption), include anxiety, agitation, restlessness, insomnia, gastrointestinal disturbances, tremors, psychomotor agitation, and, in some cases, death. Symptoms of caffeine intoxication can mimic those of anxiety and other affective disorders [2]. The cardiovascular effects include supraventricular and ventricular tachyarrhythmias. The direct cause of death is often described as ventricular fibrillation.

Generally, life-threatening caffeine overdoses entail the ingestion of caffeine-containing medications, rather than caffeinated foods or beverages [3], and have been associated with blood concentrations in excess of 80 mg/L [4].

Up to now, there has been limited detailed research regarding caffeine fatalities and there have been sporadic reports about it, although complete reviews have been published on the topic of caffeine [5,6,7]. The aim of this systematic review is to summarize data regarding caffeine lethal intoxications and try to identify possible categories at risk for it; data obtained from our study could support both clinicians and pathologists in identifying possible unrecognized cases and render possible a better and further comprehension of an ever-growing phenomenon.

## 2. Methods

### 2.1. Eligibility Criteria

The present systematic review was carried out according to the Preferred Reporting Items for Systematic Review and Meta-Analyses (PRISMA) standards [8]. Studies examining caffeine-related deaths, paying particular attention to victims of pure caffeine intoxications, were included. Study designs comprised case reports, case series, retrospective and prospective studies, letters to the editors, and reviews. The latter were downloaded to search their reference lists similarly to other papers, but yielded no other potentially eligible paper. The search was limited to human studies.

### 2.2. Search Criteria and Critical Appraisal

A systematic literature search and a critical appraisal of the collected studies were conducted. An electronic search of PubMed, Science Direct Scopus, and Excerpta Medica Database (EMBASE) from the inception of these databases to the 22th of March 2018 was performed.

Search terms were (“caffeine” OR “coffee”) AND (“toxicology” OR “death” OR “decease” OR “fatal intoxication” OR “fatality”) in title, abstract, and keywords. Cases in which death has been related to the consumption of energy drinks or caffeinated drinks were excluded because they do not represent “pure” caffeine-related deaths as they are the results of a combination of more substances such as caffeine and alcohol, or caffeine and other caffeine-like substances that may have additional mechanisms of action on cardiovascular and neurological system.

The bibliographies of all located papers were examined and cross-referenced for further relevant literature.

Methodological appraisal of each study was conducted according to the PRISMA standards, including evaluation of bias. Data collection entailed study selection and data extraction. Two researchers (D.P., S.C.) independently examined those papers whose title or abstract appeared to be relevant and selected the ones that analyzed deaths due to caffeine intoxication. Disagreements concerning eligibility between the three researchers were resolved by consensus process. No unpublished or grey literature was searched. Data extraction was performed by one investigator (M.A.) and verified by another investigator (V.F.). This study was exempt from institutional review board approval as it did not involve human subjects.

## 3. Results

### 3.1. Search Results and Included Studies

**Figure 1:**
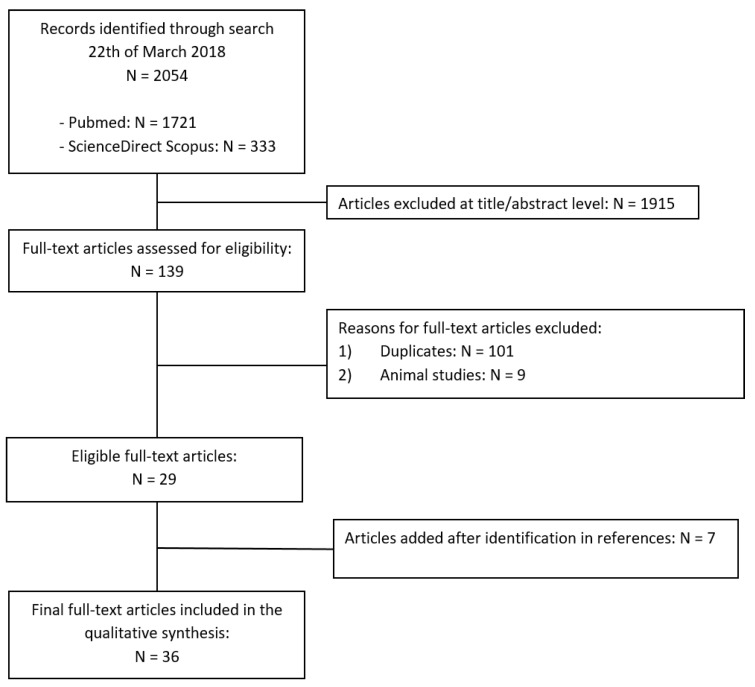
Search strategy

An appraisal based on titles and abstracts as well as a hand search of reference lists was carried out. The reference lists of all located articles were reviewed to detect still unidentified literature. Figure 1 illustrate our search strategy.

A total of 36 studies fulfilled the inclusion criteria, producing a pooled dataset of 92 individuals. The reviewed studies involved a sample size ranging from 1 (i.e., case reports) to 22 individuals (i.e., a retrospective study), with a mean of 2.6 and a median of 1, indicating skewness towards smaller samples.

### 3.2. Study Characteristics

The following data were extracted from the included studies: study source; age and sex of participants in the study; toxicological data (if reported); way of administration. An exhaustive summary of the literature, including extracted data, is shown in Table 1.

### 3.3. Risk of Bias

This systematic review has a number of strengths that include the amount and breadth of the studies, which span the globe, the hand search and scan of reference lists for the identification of all relevant studies, and a flowchart that describe in detail the study selection process. It must be noted that this review includes studies that were published in a time frame of 59 years; thus, despite our efforts to fairly evaluate the existing literature, study results should be interpreted taking into account that the accuracy of the toxicological analyses, where reported, has changed over the years.

### 3.4. Caffeine-Related Fatalities

Despite the recent policy of sale restrictions of caffeine tablets, which was introduced in 2004 in several countries, we have identified an increase in caffeine-related deaths in the last years (Table 1).

Our study allowed us to identify the manner of death as suicide (36), accidental (27), intentional poisoning (2), and uncertain (27). Routes of administration of caffeine were: oral (pills, powder, liquid) in 46 cases, intravenous in three cases, and not reported in the remaining 43 cases.

Unintentional caffeine abuse due to excessive intake of caffeine is relatively frequent and responsible for classical clinical manifestations of overstimulation. However, death due to caffeine intoxication is rare and case reports of fatalities from caffeine toxicity are relatively infrequent. We have identified 28 cases (29%), among the 92 lethal cases described in the literature, in which death was attributed to accidental causes (Table 2). The majority of fatalities were related to the ingestion of a great amount of over-the-counter caffeine products. These tend to be weight loss supplements that are frequently used and perceived as safe, but that can be toxic and linked to serious health complications.

As a result, the category of individuals consuming caffeine-containing products for dietary purposes represent a group at risk for severe intoxications, potentially leading to decease.

Despite cases where consumption of caffeine has accidentally lead to death and where caffeine was taken with suicidal purposes, we recognized three categories of individuals who have often been involved in caffeine-related deaths: athletes, psychiatric patients and infants.

In the latter group, the manner of death is linked to: intentional poisoning and child abuse; the low frequency of these categories in the other groups has encouraged us to emphasize these aspects.

### 3.5. Athletes

Five caffeine-related deaths (5%) among athletes have been described in the literature; these subjects were two amateur body builders, a basketball player and a wrestler [33,35,38]. The age ranged from 18 to 44. In all cases, the cause of death was attributed to cardiac arrest due to ventricular fibrillation.

Among these patients, body builders are well known to suffer from altered perception of body image often leading to unhealthy eating, heavy exercise habits, or even drug-taking, often with little regard to safety in spite of well publicized side effects [44]. In physiologically predisposed individuals, a combination of excessive ingestion of caffeine and strenuous physical activity can induce myocardial ischaemia by coronary vasospasm, with potentially fatal results.

### 3.6. Psychiatric Patients

Thirty-seven cases (39%) with a history of a psychiatric disorder have been identified; among the psychiatric disorders, depression is undoubtedly the most frequent (Table 3). The age ranged from 21 to 84 years-old.

The manner of death was undetermined in most of the reviewed cases, even if suicide has been recognized as the second most frequent manner. Many of these individuals have a history of past suicide attempts. A recent review on this specific topic, showed that caffeine was still a rare factor in a number of studies concerning its association with suicide attempts and death [45].

### 3.7. Infants

Fatal caffeine poisoning in children is rarely described in the literature [9,12,20,23], with the few existing cases possibly being related to child abuse and neglect, as well as accidental causes. Among accidental fatalities, iatrogenic medication errors should be taken into account.

Only two cases of intentional poisoning by using caffeine, with concomitant child abuse were reported in literature. Morrow et al. described the case of a 14-month-old child who died for caffeine intoxication [20]. Although in this case it is unknown when or how this child ingested caffeine, the clear evidence of prolonged vomiting and the high blood level of theophylline attest to a long period of severe toxicity during which no medical help was sought. These facts, as well as the delay in weight gain, chronic iron deficiency anemia, thymic involution and severe trauma to the ribs and spleen are diagnostic of child neglect and abuse.

Rivenes et al. described the case of a 5-week-old boy admitted to the hospital for evaluation of persistent tachycardia [23]. During the examination, a preliminary drug screening was negative, but a comprehensive screen subsequently performed by gas chromatographic-mass spectrometry (GC/MS) revealed the presence of high levels of caffeine, ranging from 5–12 mg/L, which are incompatible with the therapeutic values for the boy’s age. Since the source of caffeine remained unknown and its levels were far too high to be consistent with transfer from the breast milk, a referral to Child Protective Services was made. Three weeks after discharge the infant was readmitted with subarachnoid haemorrhages. He died a few dies later the admission. At the autopsy, signs of abuse, i.e., old and new rib fractures, a left spiral radial fracture, a right distal clavicular fracture, and cerebral contusion, were observed. The father admitted giving caffeine tablets to the infant “to see what it did”.

With regard to the accidental causes, only two cases of fatal caffeine intoxication are reported in the medical literature. The first, described by Di Maio et al., concerned a 5-year-old girl who ingested about 40 diuretic tablets that she found in her mother room [12]. The other one, reported by Farago, regarded a 15-month-old child who underwent a test meal in a hospital [9]. Instead of receiving 90 mL of a 2% caffeine sodium benzoate solution, the child was given 90 mL of a 20% caffeine solution (about 18 g of caffeine). Despite the prompt treatment with gastric lavage, calcium hexobarbitone, and transfusions his condition deteriorated, and he died few hours later.

## 4. Discussion

The effects of caffeine on the cardiovascular system are the result of the direct and/or indirect action of caffeine on the neuroendocrine control systems of vascular resistance, cardiac function, and electrolyte balance.

Although cases of lethal intoxication have been mainly associated with the occurrence of arrhythmic events induced by caffeine, human studies provided scarce evidence to support the substance’s ability to induce arrhythmic events in healthy subjects and in subjects predisposed to such events [2,46,47,48,49]. These findings, however, even if provided by studies differing in sample size and methods, should not be considered in disagreement with the conclusions of those studies reporting cases of lethal intoxication, as they take into account caffeine doses below the ones considered toxic for humans.

Furthermore, it should be considered that the concepts of toxic and lethal doses in humans are relative concepts, as doses below the toxic and/or lethal range may play a causal role in inducing intoxication or death. This could be due to:

- interactions with other substances with a synergistic effect when consumed with caffeine or able to increase caffeine’s blood levels;
- individuals’ pre-existing diseases and/or conditions capable of potentiating the effects of caffeine;
- inter-individual differences, mostly genetically determined, that can affect caffeine metabolism in both directions (i.e., increase or reduction), contributing to a different individual “sensibility” to the effects of the substance.

### 4.1. Caffeine and Athletes

Drug use among athletes, especially bodybuilders and weightlifters, has become a recognized problem in sports. Athletes may use drugs for therapeutic indications, for recreational or social reasons, as ergogenic aids or to mask the presence of other drugs during drug testing. Stimulants, such as caffeine, were some of the first drugs used and studied as ergogenic aids.

Sometimes, a psychiatric pathogenesis could represent the basis for excessive caffeine consumption in athletes. Indeed, some disorders are typically linked to recreational and professional athletes who consume caffeine to face fatigue and intense workouts. An example is muscle dysmorphia. This condition, also known as “reverse anorexia” or “Adonis complex”, is a subtype of body dysmorphic disorder generally affecting men, with its onset in adolescence or early adulthood, characterized by obsessiveness and compulsivity directed toward achieving a lean and muscular physique, even at the expense of health. This raises the issue of whether caffeine use causes these disorders in athletes, by inducing neuroadaptive changes within the reward neural circuit and affecting mechanisms of resilience to stress, or, vice versa, athletes with pre-morbid abnormal personalities or a history of psychiatric disorders are attracted to caffeine use, encouraged by an extrinsic motivation for exercise focused on appearance and weight control. Further studies on this topic are necessary for a full comprehension of this phenomenon.

Prior to 2004, caffeine was included in the World Anti-Doping Agency (WADA) Prohibited List of substances and methods; it was then removed, allowing athletes who compete in sports compliant with the WADA code to consume caffeine within their usual diets or for specific purposes of performance [50]. This revision was based on the acknowledgment that caffeine enhances performance at doses that are impossible to differentiate from daily caffeine use and that the practice of monitoring caffeine use via urinary concentration is not completely reliable. Despite this premise, WADA continues to measure caffeine levels through urinary concentration testing within its Monitoring Program, in order to investigate patterns of misuse of substances in sport. Differently from the WADA, the National Collegiate Athletic Association (NCAA), a non-profit association that regulates the athletes of over 1000 American institutions and associations, has a urinary concentration limit of 15 μg/mL; thus, athletes in the NCAA have to take into account that caffeine is still on the list of controlled substances.

### 4.2. Caffeine and Psychiatric Patients

Psychiatric disorders have been related to large amounts and long-term use of caffeine [51]. Furthermore, it has been suggested that caffeine may act as a trigger of psychiatric symptoms, from anxiety to depression and even psychosis [52].

In the past years, many studies have highlighted the relationship between caffeine intake and specific psychiatric disorders, in particular, bipolar [53], anxiety [54], eating disorders [55], and psychoses [56]. In addition to causing or worsening psychiatric symptoms [57], caffeine use has been investigated for its potential to interact with many psychiatric medications [58]. Caffeine is metabolized by the CYP1A2 enzyme and also acts as a competitive inhibitor of this enzyme, being able to interact with a wide range of psychiatric medications, including antidepressant, antipsychotic, antimanic, antianxiety and sedative agents. These interactions may lead to caffeine-related or medication-related side effects that may complicate psychiatric treatment, and in the most severe cases, lead to death.

With regards to alcohol use disorder (AUD), behavioural and genetic associations indicate that there is a significant link between caffeine and alcohol intake [59]. Regarding caffeine abuse by alcoholics, individuals with AUD consume approximately 30% more caffeine daily, compared to non-alcoholic individuals [60]. Besides, reports suggest that detoxified alcoholics consume large quantities of coffee following cessation of alcohol drinking, compared to their prior intake [61]. This could be a serious concern for treatment-seeking alcoholics. For example, using caffeine intake as a substitute stimulus for alcohol consumption could interfere with psychological and physiological efforts to overcome addiction-related behaviours. In addition, it is uncertain what impact a history of alcohol drinking could have on caffeine’s pharmacokinetics and metabolism profile, and whether this could affect the caffeine levels consumed by actively drinking and detoxified individuals. In conclusion, public health concern over caffeinated alcohol drinks is justified, although the nature of the caffeine/alcohol relationship is yet to be fully elucidated.

### 4.3. Caffeine and Infants

Poisoning is a severe and potentially lethal form of child abuse, and case reports have become increasingly frequent. Multiple agents have been used to poison children, including salt, water, narcotics, laxatives, diuretics, salicylates, phenothiazines, tricyclic antidepressants, insulin, sedatives and others [62,63,64].

These cases can be difficult to identify because of the clinical presentation and misleading histories. Indeed, many patients present at ages or with histories incompatible with “accidental” ingestion, others may even present with histories of recurrent illnesses suggesting previous undiagnosed poisonings.

Child abuse is considered to occur in several clinical patterns, including child neglect and physical/sexual abuse.

Generally speaking, when child neglect and abuse are carried out, other signs could be evident. For example, delay in normal weight gain and unexplained trauma are typical. In particular, intentional poisoning may be associated with other forms of abuse; approximately 20% of poisoned children may have evidence of physical abuse [65,66].

Other cases of child abuse poisoning are reported in the medical literature. Some of these are reports of deliberate parental poisoning of children and could represents the evidence of a Munchausen syndrome by proxy (MSBP), carried out by the caregiver of the child [67]. In these cases the most common mode of disease instigation involved poisoning through beverage/food contamination or subcutaneous injection [68].

The mortality rate among children diagnosed with MSBP is 9% and the most frequent causes of death are suffocation and poisoning [69]. For this reason, when child poisoning occurs, the eventual role of the caregiver as cause of poisoning must be taken into account.

Fatalities from accidental poisoning are, still nowadays, frequent in literature.

Some of these cases, as the aforementioned one, involve iatrogenic medication errors, particularly in neonatal intensive care unit where caffeine is routinely used for the treatment of the apnea [70,71]. These errors are related, in the majority of cases, to drug weighing processes.

Rivenes et al. reviewed cases of pediatric caffeine overdose and reported that the majority of cases occurred because of iatrogenic medication errors. Authors also indicated the blood levels of caffeine and highlighted that even high blood concentration of caffeine in infants can be successfully treated, thus preventing the death of the patient [23].

In conclusion when unexplained ingestions occur in children, these must be treated as non-accidental poisonings until proven otherwise. These cases required full evaluations of the social situations and sometimes required the involvement of Child Protective Services. However, caffeine toxicity could be missed because this drug is frequently not reported on routine toxicological analysis.

## 5. Conclusions

This paper represents a comprehensive review of fatal cases due to caffeine intoxication that can be found in the literature. Athletes, psychiatric patients, and infants should receive particular attention with regard to their caffeine consumption. Indeed, athletes seems to consume high quantities of caffeine as performing and image enhancing aids; at the same time, caffeine use in psychiatric patients must be considered as an important risk factor for possible intoxications because of the synergic action of caffeine with many psychiatric drugs. Finally, infants have been recognized as a last category of patients in which the use of caffeine should be completely avoided.

Indeed, previous authors have conducted systematic reviews of this topic, but they focused on specific aspects of forensic toxicology or, vice versa, took into consideration more general clinical-epidemiological issues [6,7]. Recently, a review focused on caffeine concentrations in postmortem blood in fatal cases attributed to overdose from the compound [6]. Again, a systematic review regarded the adverse effects of caffeine in pregnant women, adolescents, and children [7]. It is interesting to note how the authors hope for a change in methodology in the field of research dedicated to the use of caffeine. The topic is stressed in order to characterize the inter-individual trends, unhealthy populations, co-exposures, and outcomes, so to have a roadmap about the risk regarding caffeine-related adverse events [7].

The dangers of caffeine are related to the wide diffusion of the substance, which results in a partially conscious high consumption, due to the difficulty of ascertaining the actual amount of caffeine ingested daily and the inability to predict specific effects with regard to the “trigger role” that caffeine can have—even at “safe” doses—on underlying and not necessarily known cardiovascular conditions.

Caffeine, like alcohol and tobacco, is legally used, but, unlike the last two, its sale in the form of high concentration (e.g., powder or tablets) is not controlled or restricted.

Accidental deaths from the consumption of over-the-count and/or dietary caffeine products represent the most common cause of death in our study. The high frequency of use, the uncontrolled sales of these products, and the potentially triggering action of caffeine on cardiovascular system pose a serious risk to the health and safety of consumers.

The findings of our paper underline the importance of a fundamental principle of prevention strategy put forth by the eminent British epidemiologist Geoffrey Rose: “A large number of people exposed to a low risk is likely to produce more cases than a small number of people exposed to a high risk.”

We sincerely hope that information given about the frequency and the categories at a higher risk for caffeine intoxications may be useful for both clinicians and pathologists for a better understanding of the potentially fatal complications of coffee consumption.

## Figures and Tables

**Table 1:** Caffeine-related fatalities.

Author (Year)	Caffeine Blood Level (mg/L)	Age	Gender	Manner of Death	Route of Administration (Source)
Jokela et al. (1959) [1]	-	35	F	Accidental	Intravenous
Farago et al. (1968) [9]	1040 mg/L	15 months	-	Child abuse	Intravenous
Alstott et al. (1973) [10]	-	27	M	Suicide	Oral (pills)
Grusz-Hardy (1973) [11]	79 mg/L	45	F	Accidental	Oral (pills)
Dimaio et al. (1974) [12]	158.5 mg/L	5	F	Accidental	Oral (pills)
Turner et al. (1977) [13]	106 mg/L	34	F	Uncertain	Oral (pills)
McGee (1980) [14]	181 mg/L	19	F	Accidental	Oral (pills)
Bryant (1981) [15]	113.5 mg/L	42	F	Suicide	Oral (pills)
Chaturvedi et al. (1983) [16]	62 mg/L	21	M	Suicide	Oral (pills)
Garriott et al. (1985) [17]	129.9 mg/L	19	F	Suicide	Oral (pills)
	147 mg/L	21	M	Suicide	Oral (pills)
	343.9 mg/L	21	M	Suicide	Oral (pills)
	184.1 mg/L	23	M	Accidental	Oral (pills)
	251 mg/L	21	F	Suicide	Oral (pills)
Winek et al. (1985) [18]	240 mg/L	21	F	Suicide	Oral (pills)
Hanzlick et al. (1986) [19]	264 mg/L	44	F	suicide	Oral (pills)
	182 mg/L	20	F	accidental	Oral (pills)
Morrow (1987) [20]	117.3 mg/L	14 months	-	Child abuse	Oral (pills)
Mrvos et al. (1989) [21]	1560 mg/L	22	F	Accidental	Oral (pills)
Takayasu et al. (1993) [22]	177.0 μg/g	20	F	Suicide	Oral (pills)
Rivenes et al. (1997) [23]	117 mg/L	5 weeks	M	Child abuse	Oral (pills)
Shum et al. (1997) [24]	108 mcg/dL	15	F	Accidental	Oral (pills)
	30 mcg/dL	32	M	Accidental	Oral (pills)
Riesselmann et al. (1999) [25]	220 mg/L	19	F	Accidental	Oral (pills)
	190 mg/L	81	F	Suicide	Not reported
Watson et al. (2004) [26]	-	17	-	Suicide	Oral (pills)
Holmgren et al. (2004) [27]	173 mg/L	54	M	Uncertain	Oral (pills)
	210 mg/L	21	M	Suicide	Oral (pills)
	153 mg/L	31	M	Suicide	Oral (pills)
	200 mg/L	47	F	Uncertain	Oral (pills)
Watson et al. (2005) [28]	-	33	-	Accidental	Oral (pills)
Kerrigan et al. (2005) [29]	192 mg/L	39	F	Accidental	Intravenous
	567 mg/L	29	M	Accidental	Oral (pills)
Takeuchi et al. (2007) [30]	-	-	-	Accidental	Oral (pills)
Rudolph et al. (2010) [31]	-	21	F	Suicide	Oral (pills)
Thelander et al. (2010) [32]	90 mg/L	43	M	Uncertain	Not reported
	105 mg/L	53	M	Suicide	Not reported
	170 mg/L	47	M	Uncertain	Not reported
	86 mg/L	26	F	Uncertain	Not reported
	210 mg/L	25	F	Suicide	Not reported
	230 mg/L	40	F	Uncertain	Not reported
	210 mg/L	21	M	Suicide	Not reported
	153 mg/L	31	M	Suicide	Not reported
	173 mg/L	54	M	Uncertain	Not reported
	200 mg/L	47	F	Uncertain	Not reported
	180 mg/L	18	F	Suicide	Not reported
	166 mg/L	20	F	Suicide	Not reported
	140 mg/L	72	F	Suicide	Not reported
	80 mg/L	24	M	Suicide	Not reported
	160 mg/L	46	F	Suicide	Not reported
	113 mg/L	73	F	Uncertain	Not reported
	138 mg/L	66	M	Accidental	Not reported
	190 mg/L	84	M	Suicide	Not reported
	192 mg/L	79	F	Suicide	Not reported
	310 mg/L	33	F	Suicide	Not reported
Jabbar et al. (2013) [33]	350 mg/L	39	M	Accidental	Oral (powder)
Jantos et al. (2013) [34]	141 mg/L	25	F	Suicide	Oral (pills)
Poussel et al. (2013) [35]	190 mg/L	44	M	Accidental	Oral (pills)
Bonsignore et al. (2014) [36]	170 mg/L	3	M	Suicide	Oral (pills)
Banerjee et al. (2014) [37]	320 mg/L	50	F	Uncertain	Oral (pills)
	73 mg/L	37	F	Uncertain	Not reported
	320 mg/L	43	F	Suicide	Oral (pills)
	74 mg/L	44	M	Uncertain	Oral (pills)
	220 mg/L	57	M	Suicide	Oral (pills)
Eichner ER (2014) [38]	>70 mg/L	18	M	Accidental	Oral (powder)
Suzuki et al. (2014) [39]	179 mg/L	22 cases 20–90 years-old	-	11 unknown 7 accidental 2 suicide 2 others	
Ishikawa et al. (2015) [40]	Blood 154.2 mg/L Bile 852.3 mg/L Stomach 197.5 mg/L	20	F	Suicide	Oral (pills)
Yamamoto et al. (2015) [41]	290 mg/L	18	F	Suicide	Oral (pills)
Aknouche et al. (2017) [42]	401 mg/L	48	M	Suicide	Oral (pills)
Magdalan et al. (2017) [43]	140 mg/L	27	M	Accidental	Oral (pills)
	613 mg/L	20	F	Uncertain	Oral (powder)

**Table 2:** Accidental causes among caffeine-related deaths.

Causes	Cases
Not reported	10
Over-the-counter caffeine products	9
Errors in hospital medication	3
Drug abuse	2
Recreational use	2
Accidental ingestion by children	1

**Table 3:** Psychiatric disorders diagnosed before death.

Disease *	Number
Depression	20
Alcohol dependence	6
Sleep disorders	6
Drug dependence	4
Eating disorder	3
Panic disorder	2
Schizophrenia	2
Not specified	2
Paranoid disorder	1

* More than one disease may have been identified for each case.
